# Supplementary material for: A Neural Network Approach to Identify Left–Right Orientation of Anatomical Brain MRI
Source: Brain Behav. 2025 Feb 9;15(2):e70299. doi: 10.1002/brb3.70299 (PMC11808181; doi:10.1002/brb3.70299)
Supplement: Supplementary file 1 — Table 1. The average and standard deviation of accuracy and misclassified cases for each model created using five different seed values. Supplementary Figure 1. Overview of the skull‐stripping. OpenMAP‐T1 applied skull‐stripping to the original MRI non‐flipped and flipped images, but HD‐BET was applied only to the non‐flipped images. The 0 indicates a non‐flipped image, and the 1 indicates a flipped image. Supplementary Figure 2. Average Grad‐CAM per dataset in seed 1. Supplementary Figure 3. Average Grad‐CAM per dataset in seed 2. Supplementary Figure 4. Average Grad‐CAM per dataset in seed 3. Supplementary Figure 5. Average Grad‐CAM per dataset in seed 4. Supplementary Figure 6. Average Grad‐CAM per dataset in seed 5. [file BRB3-15-e70299-s001.docx]

**SUPPLEMENTARY MATERIALS**

Table S1. The average and standard deviation of accuracy and misclassified cases for each model were created using five different seed values.

|  |  | OpenMAP-T1 | | | | HD-BET | | | |
| --- | --- | --- | --- | --- | --- | --- | --- | --- | --- |
|  |  | Original | | Flipped | | Original | | Flipped | |
| Dataset | # subject | # failed | Accuracy (%) | # failed | Accuracy (%) | # failed | Accuracy (%) | # failed | Accuracy (%) |
| ADNI2 | 535 | 1.4 (±1.7) | 99.74 (±0.32) | 2.0 (±6.6) | 99.68 (±1.25) | 2.6 (±2.6) | 99.51 (±0.49) | 0.4 (±1.7) | 99.93 (±0.33) |
| ADNI3 | 816 | 1.6 (±2.6) | 99.80 (±0.33) | 1.6 (±2.6) | 99.80 (±0.32) | 3.6 (±5.2) | 99.56 (±0.65) | 0.8 (±0.8) | 99.90 (±0.11) |
| AIBL | 376 | 0.6 (±1.0) | 99.84 (±0.28) | 0.4 (±1.0) | 99.89 (±0.28) | 0.4 (±1.0) | 99.89 (±0.28) | 0.0 (±0.0) | 100.00 (±0.00) |
| CC359 | 359 | 3.0 (±1.6) | 99.16 (±0.47) | 2.8 (±6.0) | 99.22 (±1.68) | 5.8 (±5.5) | 98.38 (±1.53) | 2.8 (±3.8) | 99.22 (±1.07) |
| LPBA40 | 40 | 0.2 (±0.5) | 99.50 (±1.34) | 2.6 (±4.2) | 93.50 (±10.55) | 1.2 (±3.1) | 97.00 (±7.77) | 2.2 (±2.2) | 94.50 (±5.55) |
| NFBS | 125 | 0.6 (±1.7) | 99.52 (±1.37) | 0.2 (±0.8) | 99.84 (±0.70) | 0.4 (±1.0) | 99.68 (±0.83) | 3.8 (±6.0) | 100.00 (±0.00) |
| OASIS1 | 235 | 4.0 (±3.5) | 98.30 (±1.52) | 4.6 (±8.1) | 98.04 (±3.45) | 9.4 (±10.3) | 96.00 (±4.40) | 1.8 (±2.9) | 98.38 (±2.56) |
| OASIS4 | 570 | 1.4 (±2.7) | 99.75 (±0.48) | 2.8 (4.2) | 99.51 (±0.75) | 4.0 (±4.3) | 99.30 (±0.77) | 3.8 (±7.1) | 99.68 (0.52) |


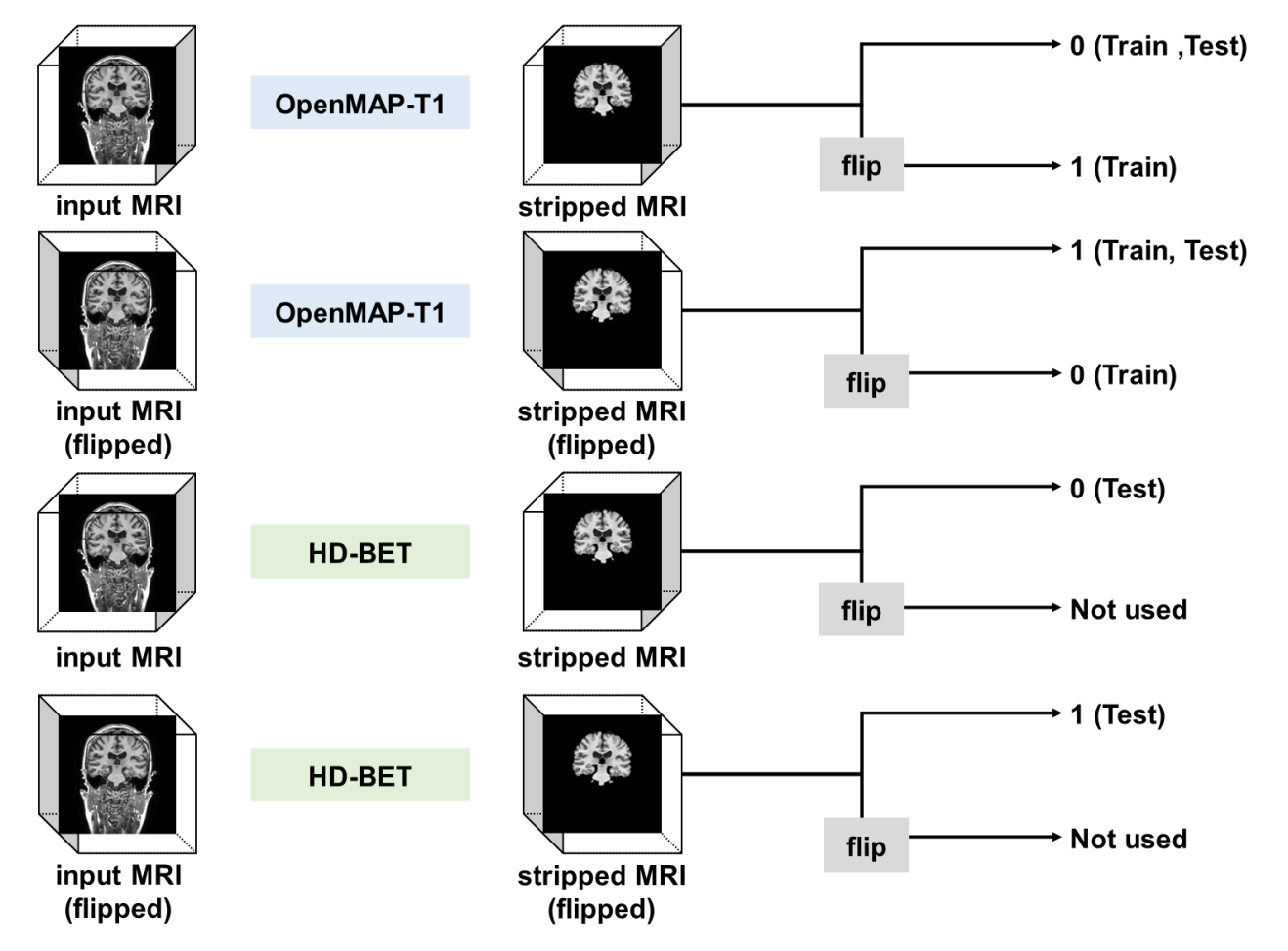


Figure S1. Overview of the skull-stripping. OpenMAP-T1 applied skull-stripping to the original MRI non-flipped and flipped images, but HD-BET was applied only to the non-flipped images. The 0 indicates a non-flipped image, and the 1 indicates a flipped image.


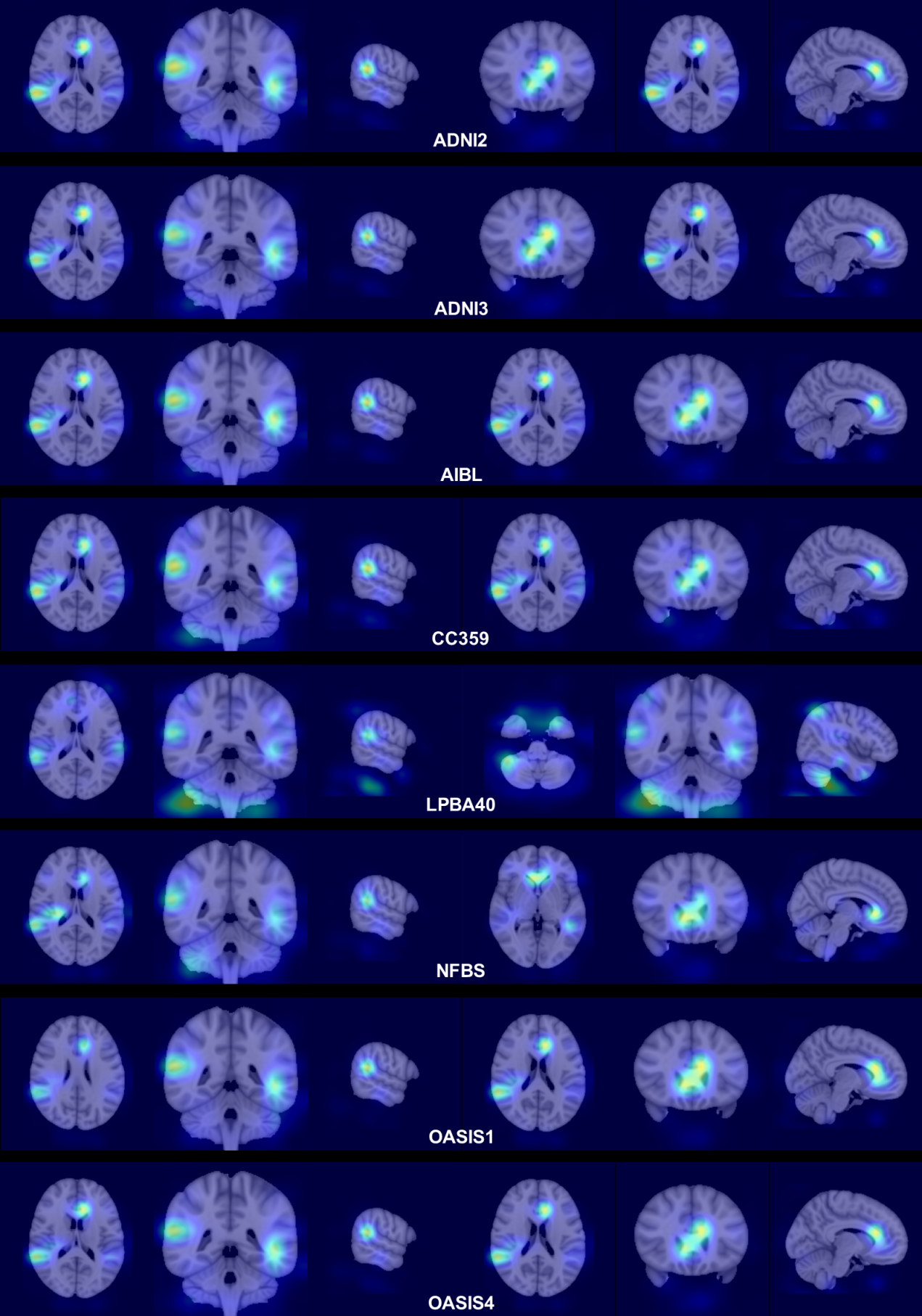


Figure S2. Average Grad-CAM per dataset in seed 1.


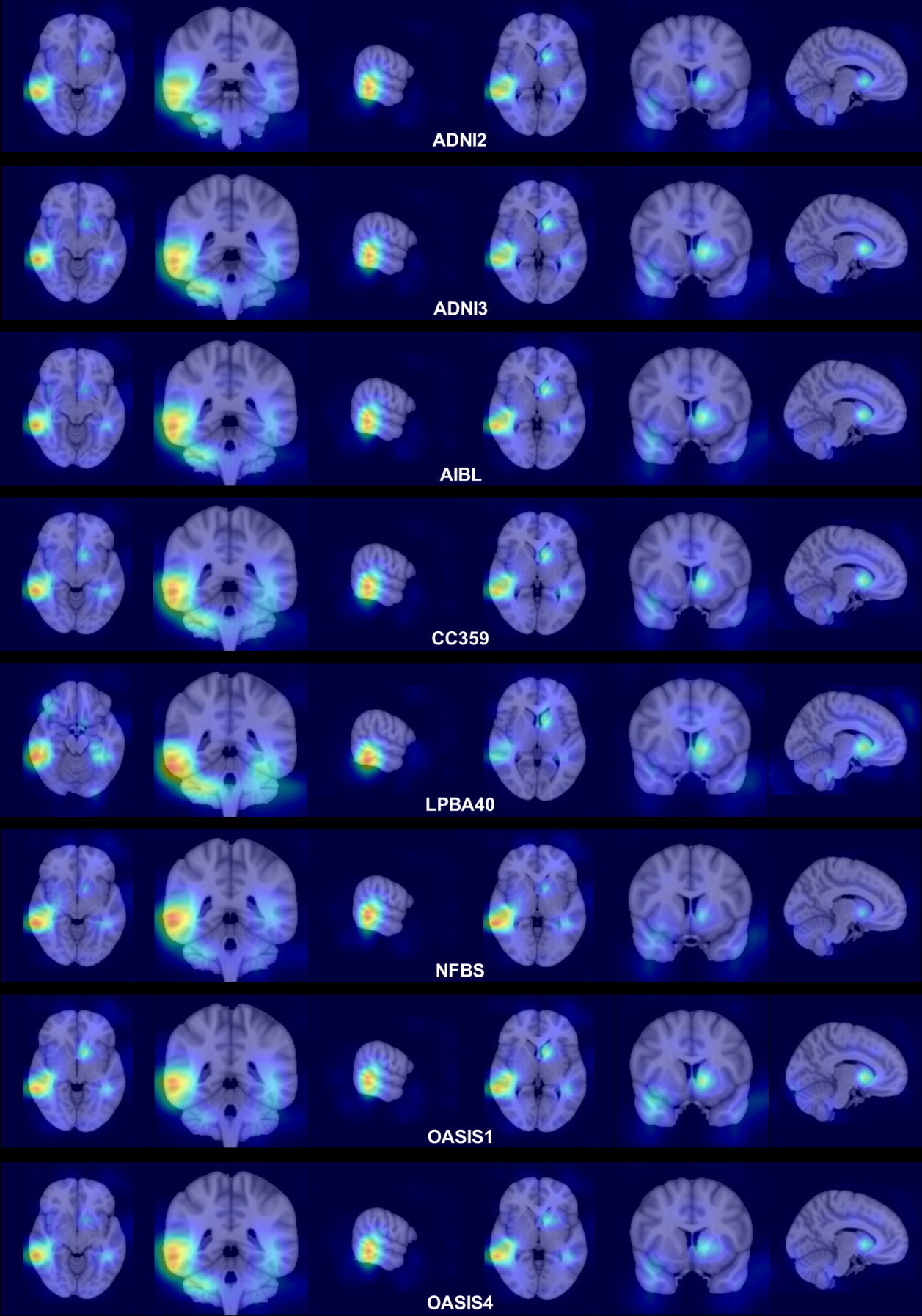


Figure S3. Average Grad-CAM per dataset in seed 2.


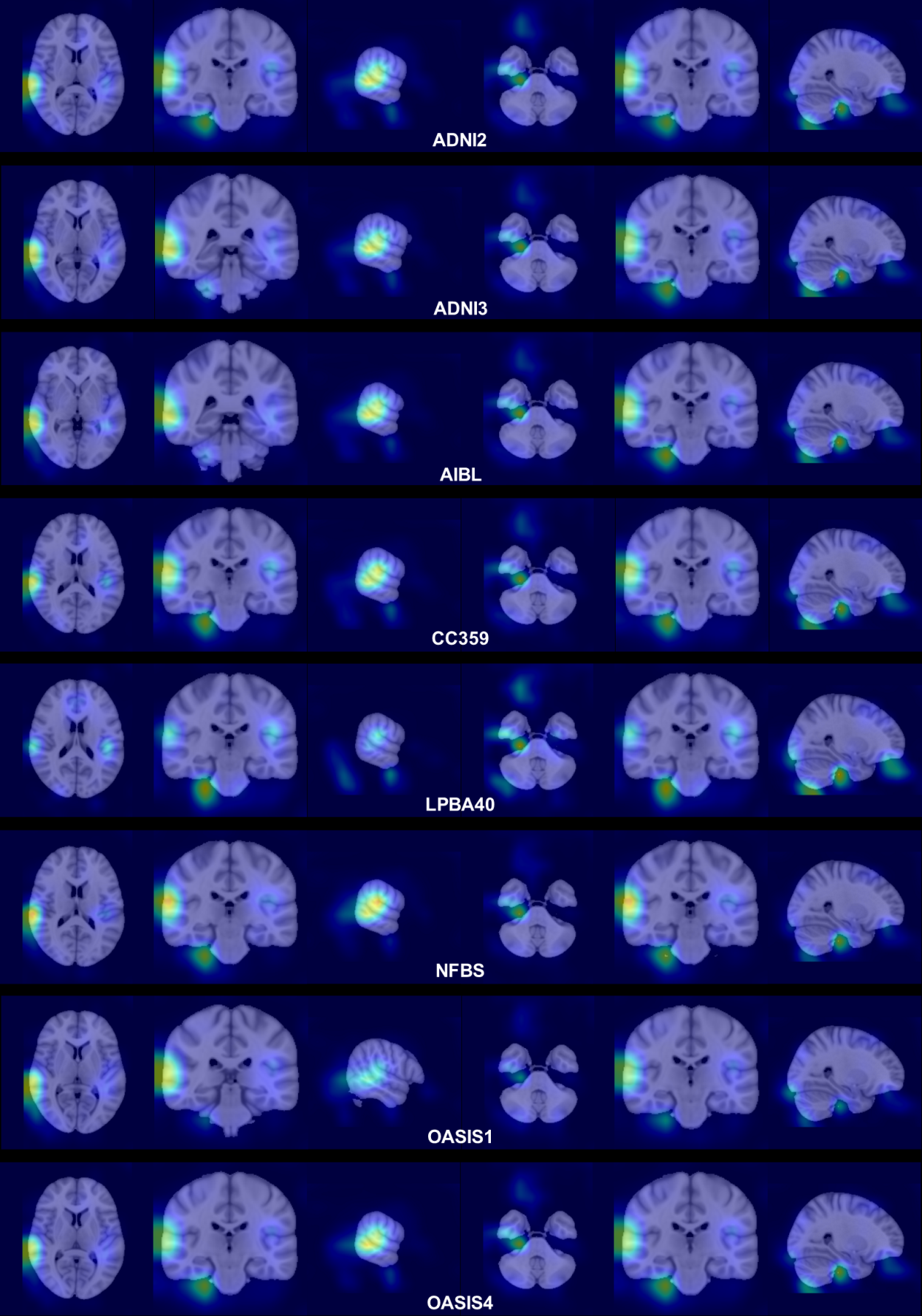


Figure S4. Average Grad-CAM per dataset in seed 3.


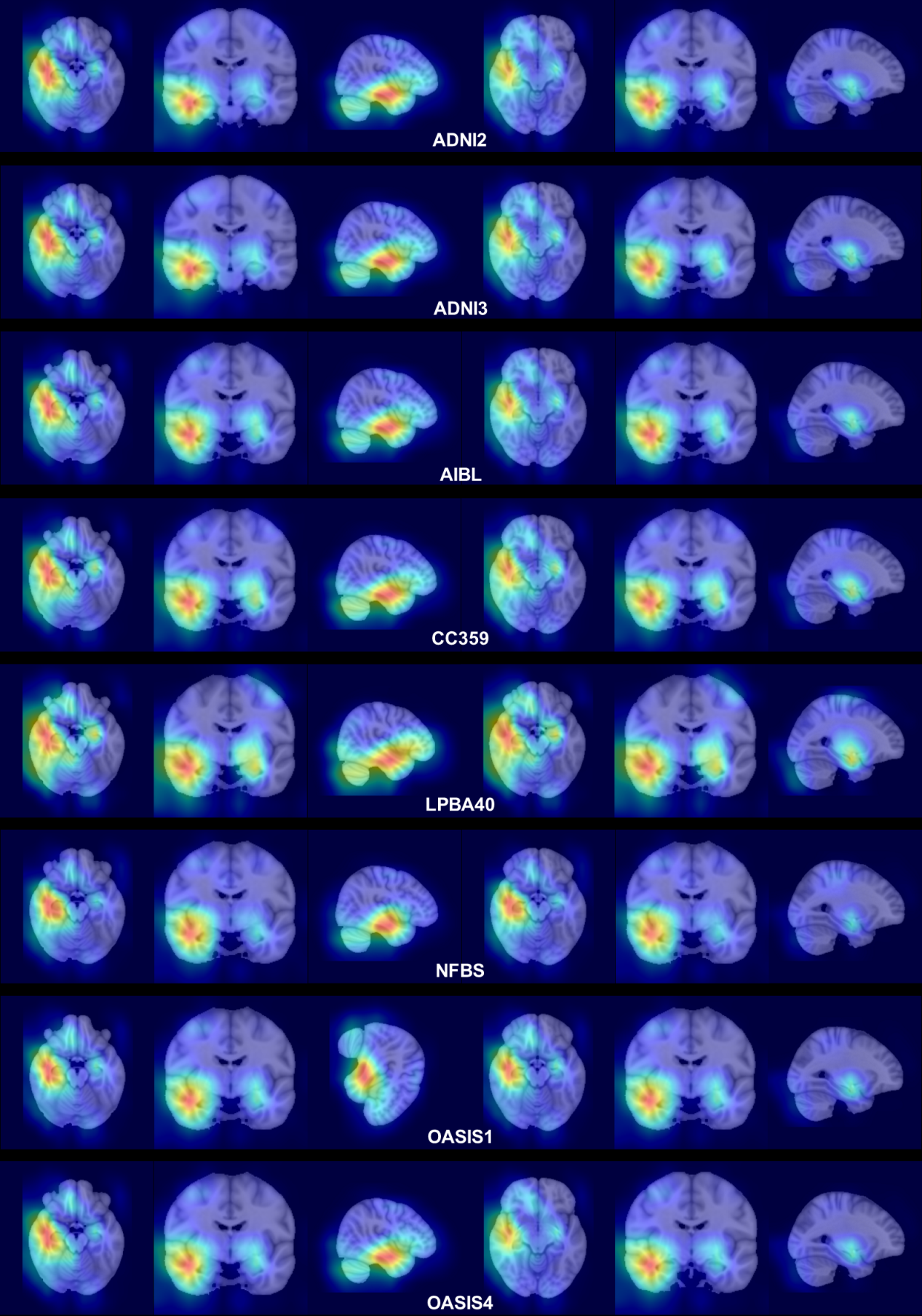


Figure S5. Average Grad-CAM per dataset in seed 4.


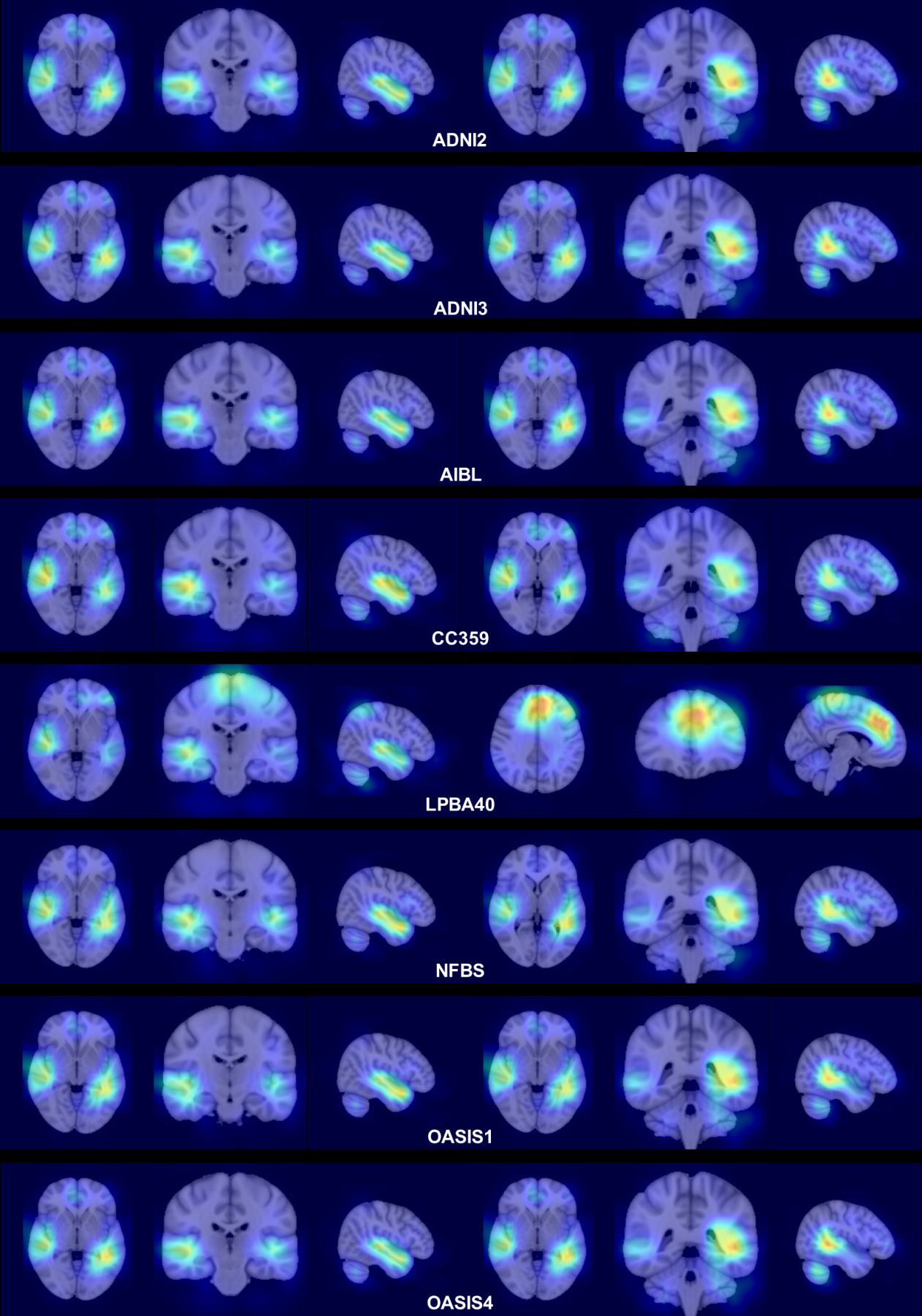


Figure S6. Average Grad-CAM per dataset in seed 5.
